# Current status and development prospects of aquatic vaccines

**DOI:** 10.3389/fimmu.2022.1040336

**Published:** 2022-11-10

**Authors:** Yang Du, Xiaoman Hu, Liang Miao, Jiong Chen

**Affiliations:** ^1^ State Key Laboratory for Managing Biotic and Chemical Threats to the Quality and Safety of Agro-products, Ningbo University, Ningbo, China; ^2^ Laboratory of Biochemistry and Molecular Biology, School of Marine Sciences, Ningbo University, Ningbo, China; ^3^ Key Laboratory of Applied Marine Biotechnology of Ministry of Education, Ningbo University, Ningbo, China; ^4^ Key Laboratory of Marine Biotechnology of Fujian Province, Institute of Oceanology, Fujian Agriculture and Forestry University, Fuzhou, China

**Keywords:** aquaculture, fish disease, fish immunity, aquatic vaccines, inactivated vaccine, liveattenuated vaccine, genetic engineering vaccine

## Abstract

Diseases are a significant impediment to aquaculture’s sustainable and healthy growth. The aquaculture industry is suffering significant financial losses as a result of the worsening water quality and increasing frequency of aquatic disease outbreaks caused by the expansion of aquaculture. Drug control, immunoprophylaxis, ecologically integrated control, etc. are the principal control strategies for fish infections. For a long time, the prevention and control of aquatic diseases have mainly relied on the use of various antibiotics and chemical drugs. However, long-term use of chemical inputs not only increases pathogenic bacteria resistance but also damages the fish and aquaculture environments, resulting in drug residues in aquatic products, severely impeding the development of the aquaculture industry. The development and use of aquatic vaccines are the safest and most effective ways to prevent aquatic animal diseases and preserve the health and sustainability of aquaculture. To give references for the development and implementation of aquatic vaccines, this study reviews the development history, types, inoculation techniques, mechanisms of action, development prospects, and challenges encountered with aquatic vaccines.

## 1 Introduction

Global aquatic products, including aquatic plants, fish, crustaceans (shrimps and crabs, etc.), molluscs (scallops, abalone, oysters, mussels, etc.) and other species (bullfrogs, jellyfish, etc.), are the third largest source of food protein for human consumption after cereals and milk, accounting for 16.4% of the total animal protein supply (1, 2). Among them, fish are the dominant species of farmed and harvested aquatic products worldwide, accounting for more than 40% of the aquaculture industry. The growing demand for fish and other aquatic foods has changed the face of fisheries and aquaculture, especially accelerating the development of aquaculture and increasing the proportion of aquaculture production to approximately 7.7% by 2020, the total fisheries and aquaculture production rises to a record high of 214 million tons, including 178 million tons of aquatic, and the proportion is expected to grow by a further 15% by 2030 (3). However, the growth of aquaculture has often come at the expense of the environment, resulting in the potential for major disease problems and food safety issues gradually increasing. Especially in recent years, viral diseases of aquatic animals such as grass carp hemorrhagic disease, carp spring virus disease, grouper iridovirus disease, infectious pancreatic necrosis, infectious spleen and kidney necrosis, infectious hematopoietic organ necrosis and viral hemorrhagic septicemia and other high incidences of disease, almost involving the main species of aquaculture, causing serious economic losses to aquaculture. It is estimated that 10% of all farmed aquatic animals die due to infectious diseases, costing more than $10 billion per year globally (4). The overall trend of aquaculture diseases shows a high incidence, diversified types, long duration of onset, a wide range of infections, and increasing year by year etc, so it is apparent that disease control in aquaculture is a matter of urgency (5, 6). Due to the lack of basic theoretical research on pharmacodynamics, pharmacogenetics, and toxicology and their impact on the aquaculture ecosystem, as well as serious problems such as resistance of aquatic pathogens, it is no longer possible to solve the current disease problems by continuing to rely on the use of various chemical drugs and antibiotics. The use of microecological preparation, immunostimulants, vaccines etc. for integrated disease control is more advocated in the new ecological farming model. Therefore, vaccination, as a green ecological control means, has become a mainstream technology for global aquatic animal disease control and has a good prospect in the development of the aquaculture industry in the world (7).

## 2 Aquatic vaccines overview

Vaccination is the most effective way to prevent and control diseases caused by viruses and bacteria. Research work on aquatic vaccines started in the 1940s. Duff et al. (1942) (8) performed the first successful oral immunization with inactivated *Bacterium salmonicida* on trout. Since the 1980s, significant progress has been made in the development of aquatic vaccines. The number of commercial vaccines available for use in fish against the major infectious bacterial and viral diseases has increased from 2 in the 1980s to over 50 currently (**Table 1**) (13, 17, 26). According to incomplete statistics, as of 2020, more than 140 aquatic vaccines have been approved worldwide (27). These include whole inactivated, peptide subunit, recombinant protein, nucleic acid, and live attenuated vaccines. However, some commercial vaccines are not always protective, and aquatic diseases continue to be severe (28, 29). Current disease vaccination is routinely used in some economically important fish species such as Atlantic salmon, Rainbow trout, Nile tilapia, Amberjack, and Striped catfish (14, 30), whereas corresponding vaccines are lacking, ineffective, or expensive in many other economic fish species. Currently, parasite vaccines face significant challenges, with only one commercial parasite vaccine available against ectoparasite sea lice. In addition, only a few trials have reported potential vaccine candidates against endoparasites (31). Furthermore, most commercial vaccines are administered *via* intraperitoneal injection (32), which may not be the best route of immunization for large-scale population vaccination and is frequently influenced by factors such as animal species, immune system status, production cycle, environment, nutrition and cost-effectiveness (33). In some countries and regions, the high cost of vaccination has led to the abandonment of vaccine defence due to widespread drug use. In light of the rapidly thriving global aquaculture industry, it is especially crucial to solve these concerns.

**Table 1:** Global commercial aquatic vaccines.

Disease	Pathogen	Major Fish Host	Vaccine Type	Antigens/Targets	Delivery Methods	Country/Region	Further Information
Viral Diseases							
Infectious hematopoietic necrosis	IHNV *Rhabdovirus*	Salmonids	DNA	G Glycoprotein	IM	Canada	https://www.dfo-mpo.gc.ca/aquaculture/rp-pr/acrdp-pcrda/projects-projets/P-07-04-010-eng.html
Infectious pancreatic necrosis	IPNV *Birnavirus*	Salmonids, sea bass, sea bream, turbot, Pacific cod	Inactivated	Inactivated IPNV	IP	Norway, Chile, UK	https://www.pharmaq.no
			Subunit	VP2 and VP3 Capsid Proteins	Oral	Canada, USA	https://www.aquavac-vaccines.com
			Subunit	VP2 Proteins	IP	Canada, Chile, Norway	http://www.msd-animal-health.no/
Infectious salmon anemia	ISAV *Orthomyxovirus*	Atlantic salmon	Inactivated	Inactivated ISAV	IP	Canada, Europe, and Latin America	http://www.pharmaq.no
Pancreatic disease virus	SAV *alphaviruses*	Salmonids	Inactivated	Inactivated SAV	IP	Norway, Chile, UK	https://www.merck-animal-health.co
Spring viremia of carp virus	SVCV *Rhabdovirus*	Carp	Subunit	G Glycoprotein	IP	Belgium	(9)
			Inactivated	Inactivated SVCV	IP	Czech Republic	(10)
Koi herpesvirus disease	KHV *Herpesvirus*	Carp	Attenuated	Attenuated KHV	IMM or IP	Israel	(9)
Infectious spleen and kidney necrosis	ISKNV *Iridovirus*	Asian seabass, grouper, Japanese yellowtail	Inactivated	Inactivated ISKNV	IP	Singapore	https://www.aquavac-vaccines.com/
Heart- and skeletal muscle inflammation (HSMI)	*Piscine orthoreovirus* (PRV)	Atlantic salmon	Inactivated	Inactivated PRV	IP	Europe	(11, 12)
Redspotted grouper nervous necrosis disease	Redspotted grouper nervous necrosis virus (RGNNV)	Sea bass	Inactivated	Inactivated RGNNV	IP	Mediterranean region	https://www.pharmaq.no/sfiles/2/54/9/file/product-info_alpha-jet_micro-1-noda_english_2018-9.pdf
Grass carp hemorrhagic disease	GCRV	grass carp	Inactivated	Inactivated GCRV	IP	China	(13)
Infectious spleen kidney necrosis, ISKN	Infectious spleen and kidney necrosis virus (ISKNV)	marine fish	Inactivated	Inactivated ISKNV	IP	China	(13)
Iridoviral disease	*Iridoviral Disease*	Red sea bream	Inactivated	Inactivated *Iridovirus*	IP	Singapore	https://www.aquavac-vaccines.com/
Koi herpes virus disease	Koi Herpes Virus (KHV)	Koi carp	Attenuated	Attenuated KHV	IMM or IP	Israel	(14)
Betanoda virus disease	Betanodavirus	Grouper	Inactivated	Inactivated Betanodavirus	IP	Japan	(15)
Grass carp haemorrhage disease	Grass Carp Hemorrhage Disease	Grass Carp	Inactivated	Inactivated Grass carp haemorrhage virus	IP	China	(13)
Viral Nervous Necrosis	Nodavirus	Seabass	Inactivated	Inactivated Nodavirus	IP	Mediterranean region	https://www.pharmaq.no/sfiles/2/54/9/file/product-info_alpha-jet_micro-1-noda_english_2018-9.pdf
Pancreas Disease	Pancreas Disease Virus	Salmonids	Inactivated	Inactivated Pancreas Disease Virus	IP	Norway, Chile, UK	https://www.merck-animal-health.co
**Bacterial diseases**							
Enteric redmouth disease (ERM)	*Yersinia ruckeri*	Salmonids	Inactivated	Inactivated *Y. ruckeri*	IMM or oral	USA, Canada, Europe	http://www.msd-animal-health.ie/products_ni_vet/aquavac-erm-oral/overview.aspx; https://www.msd-animal-health-hub.co.uk
Vibriosis	*Vibrio anguillarum;Vibrio ordalii;Vibrio salmonicida*	Salmonids, ayu, grouper, sea bass, sea bream, yellowtail, cod, halibut	Inactivated	Inactivated *Vibriosis* spp.	IP or IMM	USA, Canada, Japan, Europe, Australia	https://www.merck-animal-health.com/species/aquaculture/trout.aspx;
Furunculosis	*Aeromonas salmonicida* subsp. *salmonicida*	Salmonids	Inactivated	Inactivated *A.salmonicida* spp.	IP or IMM	USA, Canada, Chile, Europe, Australia	https://www.msd-animal-health-me.com/species/aqua.aspx
Bacterial kidney disease (BKD)	*Renibacterium salmoninarum*	Salmonids	Avirulent live culture	*Arthrobacter davidanieli*	IP	Canada, Chile, USA	(16) https://www.drugs.com/vet/renogen.html
Enteric septicemia	*Edwarsiella ictaluri*	Catfish	Inactivated	Inactivated *E. ictaluri*	IP	Vietnam	https://www.pharmaq.no/
*Edwardsiella tarda* disease	*Edwardsiella tarda*	Olive flounder	Inactivated	Inactivated *E.tarda*	IMM	Korea	(17)
	*Edwardsiella tarda*	Rainbow trout Olive flounder Turbot	Inactivated Attenuated	Inactivated *E.tarda*, Attenuated *E.tarda*	IP	China	(13)
*Vibrio alginolyticus, V. parahaemolyticus, and E. tarda* disease	*Vibrio alginolyticus, V. parahaemolyticus, and E. tarda*	Olive flounder	Multiplex antibody unique type	Anti-idiotypic antibody	IP	China	(13)
Vibrio anguillarum disease	*V. anguillarum*	Rainbow trout	Genetically engineered live vaccine	Deletion of the aroC gene	IP	China	(13)
Columnaris disease	*Flavobacterium columnaris* Arthrobacter	All freshwater finfish species, bream, bass, turbot, salmon	Attenuated	Attenuated *F. columnare*	IMM	USA	(18) https://www.drugs.com/vet/renogen.html
Pasteurellosis	*Pasteurela piscicida*	Sea bass, sea bream, sole	Inactivated	Inactivated *P. pscicida*	IMM	USA, Europe, Taiwan, Japan	ALPHA JECT 2000
Lactococciosis	*Lactococcus garviae*	Rainbow trout, Amberjack, Yellowtail	Inactivated	Inactivated *L. garviae*	IP	Spain	https://www.hipra.com/
Streptococcus infections	*Streptococcus* spp.	Tilapia, yellow tail, rainbow trout, ayu, sea bass, sea bream	Inactivated	Inactivated *S. agalactiae* (biotype 1)	IP	Taiwan Province of China, Japan, Brazil, Indonesia, Thailand	https://www.aquavac-vaccines.com/products/aquavac-strep-sa1/
				Inactivated *S. agalactiae* (biotype 2)	IP		https://www.aquavac-vaccines.com/products/aquavac-strep-sa/
				Inactivated *S. iniae*	IP or IMM		https://www.aquavac-vaccines.com/products/aquavac-strep-si/
Pasteurellosis	*Photobacterium damselae* subsp.	Sea bream, Sea bass, Amberjack, Yellowtail	Inactivated	Inactivated *Ph. damselae*	IP	Mediterranean Japan	(19)
Piscirickettsiosis	*Piscirickettsia salmonis*	Salmonids	Attenuated	Attenuated *P. salmonis*	IP	Chile	(3). https://www.pharmaq.no/products/injectable/
*Aeromonas* septicemia	*Aeromonas* spp.	Striped catfish	Inactivated	*A. hydrophila* (serotypes A and B)	IP	Vietnam	https://www.pharmaq.no/; ALPHAJECT Panga 2
Wound Disease	*Moritella viscosa*	Salmonids	Inactivated	Inactivated *M. viscosa*	IP	Norway, the UK, Ireland, Iceland	https://www.pharmaq.no
Tenacibaculosis	*Tenacibaculum maritimum*	Turbot	Inactivated	Inactivated *T. maritimum*	IP	Spain	https://www.hipra.com/
Columnaris disease	Arthrobacter	Salmonids	Attenuated	Arthrobacter davidanieli	IP	Canada	(20) (18)
Yersiniosis enteric redmouth disease (ERM)	Yersinia ruckeri Bacterin	Salmonids rainbow trout	Inactivated	Yersinia ruckeri	IMM or oral	Danish USA, Canada, Europe	(21); http://www.msd-animal-health.ie/products_ni_vet/aquavac-erm-oral/overview.aspx; https://www.msd-animal-health-hub.co.uk
Edwardsiellosis	*Edwardsiella Ictalurii*	Catfish	Inactivated	Inactivated *E. ictaluri*	IP	Vietnam	https://www.pharmaq.no/
Columnaris disease	*Flavobacterium columnare*	Channel Catfish, Salmonids, FW species	Attenuated	Attenuated *F. columnare*	IMM	USA	(18)
Vibriosis	*Listonella anguillarum*	Salmonids, seabass, yellowtail	Inactivated	Inactivated *L.anguillarum*	IMM	International	(22)
Vibriosis	*Vibrio anguillarum-salmonicida*	Salmonids	Inactivated	Inactivated *Vibrio anguillarum-salmonicida*	IMM	USA	(23)
Enteric septicemia	*Edwardsiella ictaluri*	Channel Catfish, Japanese flounder	Inactivated	Inactivated *E. ictaluri*	IP	Vietnam	https://www.pharmaq.no/
Wound Disease	*Moritella viscosa*	Salmonids	Inactivated	Inactivated *M. viscosa*	IP	Norway, the UK, Ireland, Iceland	https://www.pharmaq.no
Dropsy	Free-cell *Aeromonas hydrophila*	Indian Major Carps	Inactivated	Inactivated *A.hydrophila*	IP or IMM	Indian	(24)
	*Aeromonas hydrophila*	freshwater fish	Inactivated	Inactivated *A.hydrophila*	IP or IMM	China	(13)
Streptococcosis	*Streptococcus agalactiae*	Tilapia	Inactivated	Inactivated *S. agalactiae*	IP	Taiwan Province of China, Japan	https://www.aquavac-vaccines.com/products/aquavac-strep-sa/
Streptococciosis	*Streptococcus iniae*	Tilapia	Inactivated	Inactivated *S. iniae*	IP or IMM	Brazil, Indonesia, Thailand	https://www.aquavac-vaccines.com/products/aquavac-strep-si/
Pasteurellosis	*Pasteurella pscicida*	Salmonids	Inactivated	Inactivated *P. pscicida*	IP	Mediterranean Japan	(19)
Motile Aeromonas Septicemia	*Aeromonas hydrophila*	Salmonids	Inactivated	Inactivated *A. hydrophila*	IP or IMM	China	(13)
Flavobacteriosis	*Flavobacterium psychrophilum*	Salmonids, FW species	Inactivated	Inactivated *F. psychrophilum*	IP	UK	(25)
Lactococcosis	*Lactococcus garvieae*	Rainbow trout, yellowtail	Inactivated	Inactivated *L. garviae*	IP	Spain	https://www.hipra.com/

IM, Intramuscular injection; IP, Intraperitoneal injection; IMM, Immersion.

In recent years, with the continuous reduction of technological costs (e.g., genome sequencing, high-throughput screening of antigens, expanded cell culture, etc.), the development of novel antigen expression and delivery systems, and the massive accumulation of basic knowledge on fish mucosal immunity, aquatic vaccines are bound to see rapid development in the coming years (34). Developing highly effective mucosal vaccines and corresponding adjuvants is an important direction for future development, and optimizing vaccine delivery will undoubtedly facilitate the development of new vaccines. Aquaculture in low-income nations will be able to adopt routine immunization in the future, which will drastically reduce the use of drugs and decrease disease outbreaks and transmission on a worldwide scale. Since vaccine development processes are lengthy, it is impossible to have a vaccine on hand when a disease outbreak first starts. As a result, it is of great significance to analyze the common factors that exist among various known pathogens, find the trend rules, and conduct more clinical trials to discover vaccines with cross-protection based on existing targeted successful vaccines. In general, efficient vaccine development and utilization will contribute to the global aquaculture industry’s healthy and sustainable development.

## 3 Classification and preparation technology of aquatic vaccines

Aquatic vaccines are an effective preventative measure against many diseases and have recently gained popularity. According to the antipathogen, aquatic vaccines can be classified as bacterial, viral, and parasitic. They can also be classified according to composition, including monovalent, multivalent, or mixed (multivalent) vaccines, or classify by the preparation method, such as live, inactivated, or genetically engineered vaccines (35). It is worth noting that each type of vaccine has advantages and disadvantages and that different vaccines need to be developed and used for different pathogens and animals (as shown in **Table 2**). Aquatic vaccine production technology has developed quickly since the introduction of the *Aeromonas salmonicida* inactivated vaccine in 1942, and vaccine types have also diversified. Currently, most commercial aquatic vaccines are still inactivated, and the primary methodology for vaccine manufacture is still the inactivation of virulent wild-type pathogens by physical and chemical methods. However, inactivated vaccines have drawbacks such as short immunity duration, large inoculation dosage, adverse responses, and strict inactivation requirements. The utilization of live attenuated vaccines in aquaculture provides several benefits, including simple vaccination, low manufacturing costs, rapid delivery, low immunization dosage, and prolonged immunity. Positive outcomes have been attained with several significant economic fish. For instance, the use of antibiotics in Norwegian and UK aquaculture has significantly decreased as a result of the *Arthrobacter* Vaccine’s favourable immunological response in salmonids (12). Deficiencies are mostly caused by live attenuated vaccines’ poor safety in natural settings, which can result in virus transformation and loss of ecological environment control. It also has a limited shelf life, low thermal stability, high transport and storage requirements, and the danger of resuming mutations. Subunit vaccine keeps only the effective immunogen components and discards the irrelevant or dangerous pathogen components that trigger protective immunity. Subunit vaccines eliminate irrelevant or harmful pathogen components that induce protective immunity while retaining only the effective immunogenic components, resulting in significantly improved and stable immune effects, increased reliability, and fewer adverse reactions in the body. However, they frequently need optional adjuvants and are incapable of producing mucosal and cellular protection (36). But the plant-produced subunit vaccines are easier to manufacture, transport and store, and the immunization route is simpler and safer. They can be added to feeds as additives to induce mucosal and systemic immune responses that are more effective than traditional immunization routes and have great potential for application in aquaculture (37). Heterologous vaccines are those that are created with heterologous pathogens that cross-react with pathogens. It is safe and has a long immunity period, but it is difficult to obtain. With the advancement of immunology and molecular biology technology in recent years, there are more technical means for the development of aquatic vaccines, such as recombinant vaccine preparation technology, gene deletion attenuated vaccine preparation technology, genetic engineering live vector preparation technology, nucleic acid vaccine preparation technology, and plant vaccine preparation technology, etc. Vaccines developed using molecular biology methods provide a variety of benefits: They have immune properties that are chemically well-defined and stable; they have known chemical structures that can be engineered and modified to stimulate particular immune responses; they are free of infectious components and have no residual toxicity or risk of toxicity reversion; they can be synthesized directly or expressed through recombinant expression, which makes it easier to manufacture and enables the development of multivalent vaccines (38–40). Using genetic engineering technology to prepare vaccines is especially significant and advantageous for pathogens that cannot or are difficult to culture, pathogens with potential carcinogenic or immunopathological effects, or pathogens with poor effects or high adverse reactions to traditional vaccines. However, genetically engineered vaccines currently face varieties of challenges, including expensive development and production costs, protracted development cycles, and a single protective property. As a result, improved design strategies for the advanced, novel, and effective genetically engineered multivalent vaccines will be an important development direction for the preparation of vaccines for aquaculture.

**Table 2:** Types and characteristics of aquatic vaccines.

Vaccine type		Advantages	Disadvantages	References
Inactivated vaccine		Easy to prepare. High stability of immunogenicity. Easy storage and transportation. Easy to prepare multivalent vaccines. High safety.	Short maintenance of immunity, requiring multiple vaccinations. Induces mainly humoral immunity and cannot induce cellular or mucosal immune responses. High inoculation doses and adverse reactions. Requires strict inactivation.	(2, 32–34)
Live-attenuated vaccine		Can induce comprehensive, stable and long-lasting humoral, cellular and mucosal immune responses. Generally only one inoculation. Can be immunized by injection, immersion, oral administration and other ways.	Short validity period, poor thermal stability, and high requirements for transportation and storage conditions. A risk of reverting mutation. Relatively narrow range of use.	(9, 14, 16, 32)
Genetic engineering vaccine	Subunit vaccine	Remove harmful components, high safety. Only retain the active ingredient, the immunity is strong. High purity, and good stability. High output, can be large-scale production. Small adverse reactions.	Weak immunogenicity, need to add adjuvant, need to vaccinate several times. Cannot induce cellular and mucosal immunity.	(14, 30, 32)
	Nucleotide vaccine	Immune protection is enhanced. Simple preparation, saving time and effort. Cross-protection of homozygous strains. Safer application. Produces a durable immune response. Easy to store and transport.	DNA may induce autoimmune responses; Continuous expression of foreign antigens may produce some adverse consequences, such as tolerance. Antigen is only locally uptaken after intramuscular injection. Many factors influence the immune response. 5. A risk of foreign DNA insertion into the fish genome.	(2, 9, 13, 18)
	Living vector vaccine	Strong immune protection effect. Persistent antigen expression. Convenient immunity and less stress. Easy to expand production, low cost. It can induce mucosal immunity. It has multiple immune effects.	Foreign plasmids are easy to lose. Continuous expression of foreign antigens may produce some adverse consequences, such as tolerance. Foreign genes are easy to drift. Exogenous DNA may be integrated into the fish genome.	(2, 14, 18, 35)

### 3.1 Live-attenuated vaccine

In veterinary medicine, there are three types of live vaccines: strong, weak, and heterologous vaccines (41). Live aquatic vaccines are now more commonly used with pathogenic strains that have been weakened or mutated, such as the F25 (9) antipyretic strain of viral hemorrhagic septicemia virus (VHSV), infectious spleen and kidney necrosis virus (ISKNV), attenuated channel catfish virus (CCV) vaccine, attenuated Furunculosis vaccine, attenuated haematopoietic necrosis virus (IHNV) vaccine (42–47). The outstanding benefit of live vaccines is that the pathogen replicates in the host to produce antigenic stimuli, and the quantity, type, and location of these antigens are similar to those of natural infections. As a consequence, immunogenicity is typically strong, even without the need for a booster vaccination and the addition of adjuvants, and immunity lasts longer, displaying better immune protection (48). The live vaccine can be immunized by injection, but it is also very effective by immersion, and oral or nasal vaccination (49–52). This exceptional benefit is, however, accompanied by potential hazards, including inconveniences with storage and transportation, a short shelf life, the potential for infection to be triggered in those with low immunity, and the potential for mutations to reestablish virulence. Additionally, live attenuated/weakly virulent vaccines are excellent starting strains for vector vaccines due to their ability to retain high host invasiveness and restricted host multiplication.

There are several techniques for producing live attenuated vaccines, including chemical/physical mutagenesis, genetic engineering, attenuated culture, and antibiotic-induced attenuation. Early live attenuated vaccines were continuously transmitted to attenuated strains for screening, but the process was time-consuming and the virulence returned. Antibiotic-induced attenuation is the term for the *in vitro* screening of weak strains that are not antibiotic-dependent using media that contain antibiotics (53). Antibiotic-induced attenuated mutant strains, on the other hand, are haphazard, lack a clear genetic background, and are virulently unstable. It is difficult to ensure their safety when used in vaccine preparation and therefore hasn’t been frequently applied in following attenuated investigations. Chemical/physical mutagenesis attenuation refers to the use of chemical mutagens or altered physical conditions to induce non-situated mutations in the causative agent thereby obtaining a weakly virulent strain. The mutations produced by this induction method are random and the corresponding vaccine strain is selected due to the lack of virulence in the host animal (54, 55). Currently, with the development of genome sequencing and genetic engineering technologies, a clear understanding of the genetic background and pathogenic mechanisms of various pathogens has been achieved, and safe and efficient live attenuated vaccine strains can be constructed by knocking out virulence and metabolism-related genes of virulent strains (56). As a new means of genetic engineering, homologous recombination technology can modify DNA without any restriction endonuclease and DNA ligase. Attenuated strains constructed by knocking out one or more virulence genes using genetic engineering techniques have the advantages of a clear genetic background, weaker virulence and less susceptibility to reassortment. Some of the common virulence-related genes include housekeeping genes involved in encoding structural components of bacteria, outer membrane protein genes (57, 58), aromatic amino acid genes involved in essential metabolite synthesis (59), virulence genes involved in bacterial host resistance mechanisms, population sensing genes (60–64), iron uptake system genes (65, 66), virulence island (SPI)-related genes (67–71), secretion system-related genes, etc (72–75). Delete multiple functional genes, such as flagellin and enzymes, present more desirable immune protection (76–80), and even produce a good cross-protection (81–83).

In recent years, important progress has been made in the research of live aquatic vaccines (**Table 3**). The attenuated vaccine against *Edwardsiella tarda* and the genetically engineered weakened vaccine against *Vibrio eels* used in turbot and flounder culture have resulted in important protection of the fish (106–108). The Nile tilapia (*Oreochromis niloticus*) (109–111), silver pompano (*Pampus argenteus*) (112), giant Queensland grouper (*Epinephelus lanceolatus*) (113), Australian jewel perch (*Scortumco*) (114), bighead carp (*Aristichthys nobilis*) (115), and many other fish species can be infected by the important pathogen *Streptococcus lactis*, which threatens the aquaculture industry and causes high mortality. Liu et al. (2019) (100) developed an attenuated *S. lactis* TFJ0901 (named TFJ-ery) from a naturally low virulence *S. lactis* strain through erythromycin resistance screening, and they found TFJ-ery to be an effective attenuated vaccine candidate to protect tilapia from *S. lactis* infection. Zhang et al. (2020a) (102) developed an effective live attenuated vaccine against *S. lactis* that was also able to induce humoral and cellular immune responses in tilapia, significantly increasing the level of specific antibodies. A live attenuated vaccine was created by Liu et al. (2018) (116) to protect against the Vibrio disease in *Takifugu rubripes*, and it was discovered that the vaccination had no negative impacts on fish growth. *T. rubripes* also produced an efficient immune response after receiving the live attenuated vaccination. High-density farming of loach and deteriorating farming conditions have accelerated the outbreak of *Aeromonas hydrophila* and are a potential threat to food quality. Zhang et al. (2020) (117) developed a live attenuated vaccine to treat loach infected with *A. hydrophila* TH0426 and found that the vaccine induced an increase in enzyme activity in the serum and skin mucus of loach and upregulated immune-related genes, including IL-1β, TNF-α, IL-10 and pIgR, which effectively protected the loach from infection by *A. hydrophila* TH0426. Zhou et al. (2020) (96) developed a live attenuated vaccine against *Vibrio alginolyticus* infection that increased the expression of IgM, IL-1β, IL-6 and TNF-α and induced a protective immune response in zebrafish.

**Table 3:** Attenuated vaccine.

Disease	Pathogen	Vaccine attenuated methods	Main influencing species	Reference
Enteric septicemia	*Edwardsiella ictaluri*	Genetic engineering attenuation	Catfish	(84, 85)
		Attenuated culture		(86)
Carp disease	Carp nephritis and gill necrosis virus(CNGV)	Attenuated culture	Koi/Common carp	(87–89)
Furunculosis	*Aeromonas hydrophila*	Genetic engineering attenuation	Rainbow trout	(90, 91)
Edwardsiellosis	Edwardsiella tarda	Genetic engineering attenuation	Crucian carp	(92)
Hemorrhagic septicemia	VHSV	Genetic engineering attenuation	Olive flounder	(43, 93, 94)
*Vibriosis*	*V.anguillarum*	Genetic engineering attenuation	Duffer	(95)
	*V.alginolyticus*	Genetic engineering attenuation	Zebrafish	(96)
			Pearl gentian grouper	(97–99)
*Streptococcosis*	*Streptococcus agalactiae*	Attenuated culture	Nile tilapia	(100)
		Natural attenuation		(101)
		Genetic engineering attenuation		(102)
visceral white spot disease	*Pseudomonas plecoglossicida*	Genetic engineering attenuation	Garrupa; Yellow croaker	(64, 103, 104)
Ulcer Syndrome	*Aeromonas veronii*	Genetic engineering attenuation	loach	(105)

In general, live attenuated vaccines have great potential in the aquaculture industry because of their weakened toxicity, which cannot cause diseases, and their ability to be administered *via* natural infection routes. These vaccines provide extended and consistent antigen presentation, stimulate humoral and cell-mediated immune responses, induce host mucosal immunity, and offer the organism broad immune protection. In light of this, live attenuated vaccines have enormous promise in the aquaculture industry, and the development of novel, diverse live attenuated vaccines using genetic engineering technologies is a key area of focus for the future development of fishery vaccines.

### 3.2 Inactivated vaccine

Inactivated vaccines are vaccines that use physical or chemical methods to inactivate highly virulent pathogenic microorganisms, but they still maintain their immunogenicity and produce specific resistance in aquatic animals after vaccination. Physical inactivation methods include ultraviolet light (118), high-temperature heating (119–121), ultrasound (122) and γ-ray (123). Chemical inactivation is the process of rendering pathogenic bacteria dormant by destroying their nucleic acids or proteins with chemical chemicals. Physical inactivation is complex, costly, limiting and unstable, while in contrast, chemical inactivation is simple, low cost and reliable, and is the most commonly used method of inactivation (124, 125). In 1942, Duff first applied an inactivated vaccine against *A. salmonicida* on trout to give them immune protection, which became a precedent for fish vaccine application. An inactivated cell culture vaccine for grass carp disease was developed in China in 1986 (126), which was the first successful step in the development of aquatic vaccines. Inactivated vaccines also have limitations in that their immunogenicity is inevitably affected by the way they are prepared, which destroys the integrity of some pathogens, thus affecting the protective effect of the vaccine (127). Meanwhile, inactivated vaccines are not biologically active after entering the host and cannot colonize and reproduce, thus requiring higher doses and shorter duration of immunity, and need to be improved by adding appropriate adjuvants and making multivalent vaccines or combination vaccines. However, inactivated vaccines have the advantages of a short development period, stable storage, low cost and no virulence problems, and are currently the most reported and used vaccines in aquaculture (128).

Bacterial diseases can cause significant biological harm and thus economic losses. Although it can be controlled with antibiotics, these drugs eventually pose a threat to human health due to the development and transfer of resistance mechanisms among bacterial species (129, 130). Therefore, inactivated bacterial vaccines have been the subject to combat the spread of bacterial diseases among aquatic animals, as shown in **Table 4**, many inactivated aquatic bacterial vaccines have been reported. Liu et al. (2015) (132) used 0.5% formalin to inactivate eel vibrio at 30°C for 48 h. They found that the survival of immunized zebrafish and turbot was effectively improved with relative survival rates of (89.0 ± 4.5) % and (80.0 ± 6.9) %, respectively. Nguyen et al. (2017) (152) inactivated *Vibrio harveyi* with 0.3% formalin at 25°C for 48 h. Immunization of grouper for 6 weeks resulted in 100% relative survival and a significant increase in interleukins. Inactivation of *Haemonchus contortus* with formalin at an optimal vaccination rate of 1.0×10^8^ CFU/fish significantly promoted antibody production in turbot, and enhanced lysozyme activity, total serum protein and antimicrobial properties in the serum of immunized fish (153). Formalin-inactivated *F. harveyi* ZJ0603 vaccine (FKC) combined with β-glucan improved immune protection in pearl grouper, and the expression levels of IgM, TNF-α, MHC-Iα, IL-1β and IL-16 in the spleen and the antibody potency, lysozyme and superoxide dismutase activities in the blood of immunized fish were significantly increased (154). *Aeromonas veronii* is an extremely important infectious pathogen in carp culture. Inactivated *A. veronii* TH0426 significantly improves immune protection against crucian carp, while the addition of adjuvants can promote vaccine efficacy (139).

**Table 4:** Inactivated Bacterial vaccine.

Disease	Pathogen	Inactivation antigen	Main influencing species	Reference
Cold water *vibriosis*	*V.salmonicida*	Formalin	Atlantic salmon	(131)
*Vibriosis*	*V.anguillarum*	Formaldehyde/Formalin	Zebrafish; Scophthalmus maximus; Flounder	(132, 133)
	*V.harveyi; V.mimicus; V.alginolyticus*	Formalin	Garrupa; Pearl gentian grouper; Zebrafish; Grass carp; Large yellow croaker	(134–137)
Ulcer syndrome; ascites disease	*aeromonas veronii; Edwardsiella ictaluri*	Formalin/Formaldehyde	Yellow-head catfish; Crucian carp; Carp	(138–140)
*Tenacibaculosis*	*Tenacibaculum finnmarkense*	Formaldehyde	Salmon	(141)
*Francisellosis*	*Francisella noatunensis* subsp. *orientalis*	Inactivated adjuvants: Montanide	Nile tilapia	(142)
Crack-head disease	*Edwardsiella ictaluri*	Formalin	Red Sea Bream	(143)
		Formaldehyde	Yellow-head catfish	(144)
Edwardsiellosis	*Edwardsiella tarda*	Formalin	Flounder	(145)
*streptococcosis*	*streptococcus*	Formalin	Olive flounder; Garrupa	(124, 146)
	*streptococcus agalactiae*	Hydrogen peroxide	Nile tilapia	(147)
*lactococcosis*	*Lactococcus garvieae*	Formalin	Grey mullet fish; Rainbow trout	(148, 149)
Furunculosis	*Aeromonas salmonicida*	Formalin	Rainbow trout	(150)
Hemorrhagic septicemia	*Aeromonas hydrophila*	Sonication	Grass carp	(151)

Inactivated viral vaccines are safe and won’t cause any infections because the virus has lost its activity, many inactivated aquatic viral vaccines have been reported in studies (**Table 5**). However, the immune response is lessened or the duration of immunity is reduced since the virus is only a fragment and not an intact virus (173). It has been reported that the infectious hematopoietic necrosis virus (IHNV) vaccine inactivated by 1.5 mmol/L Beinylating agents can significantly improve the survival rate of rainbow trout (155). Neural necrosis virus vaccine inactivated by 4 mmol/L BEI can not only significantly promote the expression of various immune genes in grouper, but also cause body fluids and cellular immunity (162). Inactivated cyprinid herpesvirus type 2 (CyHV-2) vaccine with 0.1% BPL (chemical formula C3H4O2) at 4°C significantly induced non-specific and specific antiviral immune responses in carp, resulting in relative immune protection of 71.4% (164). Grass carp had a relative immune protection rate of more than 80% after receiving the grass carp reovirus (GCRV) vaccine inactivated with 0.1% BPL (165). The addition of adjuvants is effective in improving the effectiveness of inactivated viral vaccines, for example, because squalene and aluminium hydroxide adjuvants prolong and improve the efficiency of formalin-inactivated VHSV in flounder, which can be controlled by a single dose of this vaccine administered before winter (174). Montanide IMS 1312 adjuvant significantly improves the immune efficiency of formaldehyde and β-propiolactone inactivated tilapia lake virus-inactivated vaccine and increases protection against tilapia (175).

**Table 5:** Inactivated Viral vaccine.

Disease	Pathogen	Genome	Inactivation antigen	Main influencing species	Reference
infectious hematopoietic necrosis	Inactivated infectious hematopoietic necrosis virus (IHNV)	A single strand of RNA	BEI; BPL; Formaldehyde	Rainbow trout	(155)
Pancreas disease	*Salmonid alphavirus*	A single strand of RNA	Formalin	Salmon	(156, 157)
Viral hemorrhagic septicemia (VHS)	VHS virus (VHSV)	ssRNA	Formalin	Olive flounder	(151, 158)
viral nervous necrosis disease	Nervous necrosis virus(NNV)	A single strand of RNA	Formalin/BEI	Garrupa; Senegalese sole	(159–162)
giant salamander Iridoviruses disease	giant salamander Iridoviruses	DNA	BPL	Chinese giant salamander	(163)
Herpesviral haematopoietic necrosis (HVHN)	cyprinid herpesvirus 2(CyHV-2)	Double-stranded DNA	BPL	gibel carp	(164)
hemorrhagic disease	Grass carp reovirus	dsRNA	BPL	Grass carp	(165)
viral nervous necrosis disease; grouper iridoviral disease	viral nervous necrosis virus(VNNV); grouper iridovirus (GIV)	single strand of RNA; DNA	BEI	orange-spotted groupers	(166)
viral encephalopathy; retinopathy (VER)	Nodavirus	A single strand of RNA	Formalin	European sea bass	(167–169)
			UV		
White Spot Disease (WSD)	white spot syndrome virus (WSSV)	Double-stranded DNA	Formalin	*Penaeus monodon*	(170)
Tilapia virus disease	Tilapia lake virus(TiLV)	A single strand of RNA	Formaldehyde; BPL; Heat	Nile tilapia	(171, 172)

### 3.3 Genetically engineered vaccine

Genetic engineering vaccines primarily use genetic engineering technology to isolate immune antigen genes from bacteria or viruses and transfer them into animal cells or recipient organisms, allowing the organism to produce a large number of protective antigens and improve disease resistance. The main types include recombinant subunit vaccines, nucleic acid vaccines, recombinant live vector vaccines, gene deletion/mutation vaccines and plant vaccines. Aquatic vaccines have been widely used in aquaculture since the 1980s, but the majority of commercial vaccines are inactivated bacterial vaccines, with only a few being genetically engineered recombinant vaccines.

#### 3.3.1 Subunit vaccine

A subunit vaccine is a vaccine prepared by encoding a pathogen antigen into a recombinant expression vector and expressing a gene product (recombinant peptide or recombinant protein) using a prokaryotic or eukaryotic expression system. Subunit vaccines utilize only the antigenic component for vaccination and pose no risk of causing disease in the host or non-target species because subunit vaccines cannot replicate in the host. Furthermore, subunit vaccines can be manufactured in a highly characterized state, they can target immune responses to specific microbial determinants, they can incorporate non-natural components, and they can be freeze-dried, allowing for non-refrigerated transport and storage. Heterologously expressed recombinant subunit vaccines address both difficulties with antigen supply and vaccination safety, are easily accessible when testing is required and can be produced and used consistently. Some of the most important characteristics of vaccine candidate molecules are that they are highly conserved among different strains of the same species and that they are expressed on the pathogen’s surface so that antigen-presenting cells can easily recognize them due to their immunogenicity. The high immunogenicity of outer membrane proteins (OMP) is considered a promising candidate for the design of antimicrobial drugs and vaccines in various pathogenic strains. Numerous other studies have reported that outer membrane proteins (OMPs) are generally very immunogenic due to their exposed epitopes on the cell surface (176) and have the potential of a vaccine candidate for fish against *V. anguillarum* (177, 178), *Vibrio mimicus* (179), *V. harveyi* (180), *Vibrio ichthyoenteri* (181), *P. olivaceus* (182), *E. tarda* (183) and *A. hydrophila* (184) infection. Flagellin is also well-studied and effective subunit vaccine candidate (185). Viral capsid proteins are also regarded as significant possible antigens for the development of subunit vaccines (186, 187). It is concerning that subunit vaccines frequently contain a single antigen, resulting in low immunization efficiency, necessitating multiple immunizations and the addition of adjuvants to improve the immunization effect or the development of multiple vaccines (188–192). Cytokines can act as signalling molecules involved in the immune system and as low molecular weight glycoproteins or peptides regulating the host defence network (193). Compared to the most commonly used adjuvants in fish culture (e.g., aluminium plate adjuvants and oil adjuvants), cytokines have advantages as immune adjuvants in initiating the expression of co-stimulatory molecules and polarization of antigen-presenting cells (194). A large number of cytokines have been identified in various fish species, for example, IL-1β, IL-8, G-CS, TNF-α, IL-6, IL-2 and other cytokines have been found to enhance the immune effect of subunits in flounder (195, 196).

Subunit antigens have been produced in bacteria, yeast, transgenic plants, insects, mammalian cell cultures, and cell-free platforms. Among them, bacteria can easily and abundantly express antigens, but cannot post-translationally modify the expression products; therefore, eukaryotic antigens are usually not expressed in bacteria. And the expressed exogenous proteins have damaging effects on the host bacteria, and there may be a risk of endotoxin contamination in the expression end products. Yeast is widely used in food, beverage and feed production, and its genetic and biochemical background is clear. Yeast not only expresses proteins in a secreted form but also post-translationally modifies them. Yeast is easy to operate, has a fast growth rate, and has now mastered a lot of experience in large-scale fermentation production, with great potential in aquatic vaccines. The advantage of the mammalian expression system is that it can post-translationally modify the expression products close to the natural conformation, which is important for inducing immune responses to vaccine antigens. However, the cellular production of vaccines is too costly and potentially carries the risk of contamination with foreign or oncogenic components (2, 197). The plant-based vaccine also referred to as a transgenic plant vaccine, involves the introduction of antigen genes into plant cells and their expression, or the replication of antigen genes in plants using pathogenic vectors to produce high yields of the target protein. Transgenic plants are currently being used to produce vaccines against viruses, bacteria, and parasites, and clinical studies have shown that plant-produced recombinant drug proteins are safe and effective (198, 199). Compared to microbial or mammalian cell expression, plants have a higher biosynthetic capacity of which plant seeds, tubers and fruits are excellent locations for protein aggregation and conservation. Stably expressed plants integrating antigenic genes can be produced in large quantities by asexual or sexual reproduction. They can also undergo post-translational modifications like glycosylation and complex folding and assembly to improve vaccine immunogenicity (200). Furthermore, there are no safety problems with plant-made subunit vaccines compared to live vaccines, and no harmful substances like bacterial endotoxins or highly glycosylated proteins from yeast have been discovered in plant production systems (201). Plant-produced recombinant subunit vaccines can also deliver multiple antigenic proteins simultaneously (202). Thus, plant-based platforms offer appealing benefits for recombinant subunit vaccines. Plant-produced vaccines are appropriate for oral delivery, as the vaccine antigen is absorbed by M cells in the epithelium after passing through the stomach and into the intestine, eliciting mucosal and systemic immune responses (203). Aquatic vaccines made from edible plants hold great promise for oral vaccines in aquaculture, as such transgenic plants can be directly crushed and added to feeds as additives to trigger a particular immune response. The best plants for edible vaccines include vegetables and fruits that have been shown to generate substantial amounts of exogenous proteins, such as lettuce, tomatoes, potatoes, rice, and cabbage (204, 205). According to a study, feeding striped bream transgenic rice healing tissue with recombinantly expressed Iridovirus coat protein (MCP) dramatically increased the fish’s disease resistance (206).

Microalgae are natural baits and nutritional additives for aquatic animals, especially for the small fry, and are not only rich in proteins, lipids and essential nutrients and potential immunogenicity, but they also have a shorter culture cycle than other macrophytes to complete complex protein folding to form active recombinant exogenous protein, making them an ideal system for aquatic vaccines production and oral delivery (207–209). *Chlamydomonas reinhardtii*, *Dunaliella salina*, and cyanobacteria have been reported to express antigenic genes for the prevention and control of infectious diseases. For example, oral administration of *D. salina* and *C. reinhardtii* integrated with VP28 improved the survival of shrimp exposed to WSSV (210, 211). Feeds prepared with *C. reinhardtii* expressing Antiviral double-stranded (ds) RNA-YHV increased the survival of shrimp by 22% after a yellow head virus (YHV) attack (212). Through successful expression in *Nicotiana benthamiana*, Atlantic cod neuro necrosis virus (ACNNV) VLPs be effective in protecting sea bass from viral attack (213). Diatom recombinantly expressed heat shock protein (GroEL) and outer membrane protein (IglC) of *Francisella orientalis* (Fo), both of which enhance the immune response of Nile tilapia and improve immune protection in fish (214). To date, however, many microalgae have not become an effective and established genetic manipulation system, and no plant-produced aquatic vaccines have been commercialized (215). Therefore, further research on the production of aquatic vaccines using plant biotechnology is essential. To improve the activity of vaccines and heighten the level of induced animal immune responses, research on plant-produced vaccines is currently concentrated on increasing the concentration and purity of antigens in transgenic plants as well as addressing shortcomings in the ambiguous efficiency of modifications, such as glycosylation, methylation, and polymerization (216). In addition, animal species, animal diet, disease type, vaccine characteristics, production cycle, production scale, biosafety issues, future commercial prospects, and management difficulties need to be considered before selecting a plant expression system for fish vaccine production (5).

Overall, subunit vaccines offer a wide range of uses and are a good option for some hard-to-culture microorganisms. They can be used to protect both homologous pathogens and multiple infections by combining the antigenic genes of different pathogens. The effectiveness of subunit vaccinations to generate mucosal and cellular immunity in vertebrate hosts has significantly improved even though they are less immunogenic than inactivated vaccines due to ongoing advancements in antigen delivery and vaccine adjuvants. Subunit vaccines are currently plagued by the difficulty of expressing recombinant viral and protozoan membrane antigens in their natural structural state, as well as the production of misfolded or misprocessed proteins by microbial systems (particularly *E. coli* and yeast) that lack the conformational epitopes required to produce protective antibodies in the host. Furthermore, the fact that subunit vaccines are primarily administered by injection and the need for adjuvants raises the cost of their use.

#### 3.3.2 Nucleic acid vaccine

Nucleic acid vaccine, which includes DNA and RNA vaccines, refers to the introduction of plasmids containing a gene encoding an antigen protein into the host to express the antigen protein through the host cell and induce the host cell to produce an immune response to the antigen protein to achieve immune effects. In 1996, Anderson et al. (1996) (217) prepared a DNA vaccine pCMV4-G containing the IHNV G gene and immunized trout by intramuscular injection, which improved trout resistance, launching research into the use of nucleic acid vaccines for fish. After traditional vaccines and genetically engineered subunit vaccines, nucleic acid vaccines have emerged as a hot spot for aquatic vaccine research and development. Although nucleic acid vaccines provide enhanced immune protection, are simple to prepare, save time and effort saving, are safer in an application, and have a long duration of an immune response, they are prone to immune tolerance and have a low probability of plasmid DNA integration into the host genome and causing autoimmune diseases and insertional mutations. Nucleic acid vaccines are frequently very effective in preventing viral infections, such as fish bouncing viruses (rhabdovirus), because they use the same cellular mechanisms that viruses use when they enter the host cell (218, 219). Huo et al. (2020) (220) developed recombinant plasmids pcORF25 and pcCCL35.2 to serve as a vaccine and molecular adjuvant against CyHV-2, respectively. They discovered that pcORF25/pcCCL35.2 effectively increased mRNA expression of critical immune genes (IL-1, IL-2, IFN-γ2, and viperin) and significantly inhibited CyHV-2 replication in the head kidney and spleen tissues. DNA vaccines are also effective in preventing bacterial infection in fish, such as *Vibrio anguillarum* (221–223), *E. tarda* (224), *V. harveyi* (225) and so on. Several DNA vaccines have been approved for marketing, and the first licensed DNA vaccine ever was a DNA vaccination for IHNV that was approved in Colombia, UK, in 2003 (226), and was approved for marketing in Canada in 2005 (218). In 2018, a DNA vaccine against the Salmon pancreatic disease virus (SPDV) was approved for marketing in Norway and the EU (227). A commercially available vaccine based on DNA plasmid has been authorized to be used in Norwegian aquaculture against Pancreas disease (PD) caused by salmonid alphavirus subtype 3 (SAV3) in Atlantic salmon SAV3 in salmon since 2018 (228, 229). Studies on DNA vaccines against parasitic fish diseases have also been reported, for example, immunization of obliquely banded grouper with DNA vaccines prepared by stimulation of *Cryptococcus repressor* antigens improved the resistance of the fish (230). Intramuscular injection of DNA vaccine against the parasite Ichthyophthirius multifiliis (Ich) induced significant upregulation of immune genes in the head kidney of catfish, including IgM, CD4, MHCI, TCR-α, IFN-γ, complement component 3 (C3) and Toll-like receptor (TLR), and the fish produced anti-Ich antibodies, obtaining a higher immunoprotective effect (231). A DNA vaccine against *Ichthyophthirius multifiliis* was also effective in immunizing channel catfish (232).

Compared to conventional vaccines, DNA vaccines are more stable, safer, and largely free of toxicity reversal. However, DNA vaccines are not mature enough and, at present, it is not possible to determine whether the host genome will integrate with exogenous DNA to disrupt the genetic homeostasis in the host. What’s more, DNA vaccines have potential side effects, including immune tolerance of the organism to the expressed antigen, chromosomal integration, and injection site inflammation (219). RNA vaccines have received significant attention in recent years, and they have many advantages. For example, RNA is not infectious, it can be degraded by normal cellular processes, and there is no potential risk of infection or insertional mutagenesis. Therefore, RNA vaccines have a promising future in aquatic vaccines (233).

#### 3.3.3 Recombinant live vector vaccine

A live vector vaccine is a genetically modified vaccine that uses a non-pathogenic virus or bacteria as a carrier to express protective immune-related antigens and deliver the antigens to the lymphoid tissue of the intestine, generating intestinal mucosal immune responses and system immunity. It is characterized by the combination of the high immunogenicity of live attenuated vaccines and the precision of subunit vaccines. A significant benefit of the live vector vaccines is their ability to effectively induce cellular and humoral immunization and even mucosal immunization by inducing target antigen expression *in vivo*, resulting in endogenous antigen processing and MHC class I restriction antigen presentation. Second, the production cost of live vector vaccine is low and the risk of virulence is low. The antigen genes of different pathogens can be inserted into the vector and expressed simultaneously, thus achieving the goal of preventing multiple diseases with one needle, providing the possibility for the development of multiple or multiple vaccines (234). At present, bacterial live-carrier vaccines have been studied in fish live-carrier vaccines, such as *E. coli*, *E. tarda*, Salmonella, Listeria, Lactobacillus, *Bacillus subtilis*, etc. have been reported. It is worth noting that with the in-depth research on the function of gut microbes in recent years, the role of probiotics has been paid more and more attention. Probiotics are safe and non-toxic, can promote the intestinal absorption of nutrients better, enhance the body’s immunity, and also have a good adjuvant effect as a live vaccine carrier. Studies have shown that recombinant live vector vaccines constructed with *Lactococcus lactis* and *B. subtilis* can elicit a protective immune response in the host (235–238). Oral administration of *Lactobacillus casei* CC16 strain expressing the flaB and OmpAI antigens of *A. veronii* significantly induced carp-specific antibody responses and carp-specific antibody responses, up-regulated IL-10, IL-β, IFN-γ, TNF-α and other immune genes expression, and improved the fish immune protection (239, 240). The vectors of live viral vector vaccines are generally attenuated strains, and exogenous antigens are inserted into non-essential regions of the vector genome to form a new recombinant virus, with the exogenous genes being expressed concurrently with vector replication. Baculovirus (BmNPV), a pathogen of silkworm (*Bombyx mori*), can be used as a potential vaccine vector for clinical application (241). Baculovirus (Baculovirus) and IHNV can both be used as vaccine carriers to induce effective immune protection of the host (242, 243). It has been reported that the recombinant BacCarassius-D4ORFs-BmNPV vector vaccine can effectively protect crucian carp from CyHV-2 infection, with relative survival rates of 59.3% and 80.01% by oral or injection vaccination, respectively (244).

Although live vector vaccines have many advantages in terms of immunization mode, strain culture, and gene delivery, there are still many drawbacks, such as the fact that some bacteria with reduced virulence can still be harmful to the body’s health and that long-term immunity causes the body to become tolerant to producing antigen, which hurts the immune system. Therefore, further optimization of vector selection and immunization strategy is needed to improve the protective effect of live vector vaccines.

## 4 Aquatic vaccination methods

The development of protective vaccines and their proper use is essential for successful vaccination. Vaccination should be administered before pathogen exposure to allow sufficient time for immunity to develop. The route of immunization used is also selective, depending on the type of vaccine, cost, type, size, and several fish to be immunized, as well as the purpose of immunization. In aquaculture, three vaccination methods are commonly used: injection, immersion, and oral immunization (245). As shown in **Table 6**, each immunization method has advantages and disadvantages, and in practice, we must select the best vaccination method based on the characteristics of the vaccine, the type of animal, etc.

**Table 6:** Comparison of vaccination methods.

Inoculation regimes		Feature	Advantages	Disadvantages	Reference
injection		Can be done manually or automatically. A small amount of known antigen can be directly transmitted to fish, which plays effective protection and long protection time.	A delivery method with the best protective effect is the only choice for adjuvant vaccines. A small amount of use can be sustained protection.	If anaesthesia is not performed, it usually causes a large animal stress response; needs a lot of labour and is high cost.	(153, 246, 247)
immersion	direct immersion (DI)	Transfer fish to water containing vaccines for some time and put them back in their tanks.	Easy to operate; suitable for immunization of small-sized fish species; better effect; less stressful effect	Can only be applied to concentrated fish stocks; requires booster measures; high vaccine dosage; no versatility.	(248, 249)
	hypertonic immersion (HI)	Immerse the fish in a solution such as urea or sodium chloride for a short time, and then soak in the vaccine.			
	spray vaccination	Mainly for larger individuals.			
Oral		Vaccines are made into baits and fed to activate intestinal mucosal immunity; the best vaccination method for fish and other aquaculture animals.	No stress effect; suitable for dispersed fish; not constrained by the size of the fish; simple and easy to operate.	The vaccine is easily destroyed by the digestive tract; poor immunization effect; high vaccine dosage.	(236, 250, 251)

### 4.1 Injection immunization

The most effective method of vaccination is injectable immunization. It ensures that each immunized fish receives an equal amount of vaccine to stimulate the body’s immune response, allowing the immunized fish to achieve long-term and stable immune protection. This vaccination method requires fewer vaccines and is especially appropriate for inactivated, subunit, and nucleic acid vaccines, as well as mixed, monovalent, and multivalent vaccines. However, there are numerous issues with the injection immunization process, such as the difficulty of handling and, in particular, the inconvenience of immunizing small fish under 20g, but fish smaller than this are generally the most susceptible to disease. It can also harm the fish mechanically, causing ulceration at the injection site and adhesions between internal organs and the peritoneal wall. Second, the handling, anaesthesia, and injection processes not only stress the fish but also increase labour costs, particularly when immunizing large numbers of fish. Machine injection, as opposed to manual injection, can reduce stress on fish, reduce labour intensity, shorten injection time, and overcome some disadvantages of the injection immunization method itself (252). Many patented inventions of vaccination apparatus are now commercially available, which not only greatly facilitates large-scale vaccination operations by farmers, but also improves the efficiency of vaccine utilization and facilitates the vaccine development process. Injectable immunizations typically include intraperitoneal and intramuscular injections. Inactivated vaccines are frequently administered intraperitoneally, subunit vaccines usually require adjuvants, and some nucleic acid vaccines are only suitable for intramuscular immunization due to their high uptake by muscle tissue, which can induce a systemic immune response. Corbeil et al. (2000) (253) evaluated the vaccination effect *via* various vaccination routes including intramuscular injection, scarification of the skin, intraperitoneal injection, intrabuccal administration, gene gun, or immersion with pIHNVw-G, and found only the intramuscular injection and using gene gun induced protective immunity in Rainbow trout fry. The pSAV/HE DNA vaccine elicited a strong immune response at the injection site after intramuscular injection and provided high protection in Salmon, whereas neither the pSAV/HE alone nor the mixed inactivated low-dose virus vaccine by intraperitoneal injection elicited an immune response in *Caenorhabditis elegans* or improved the effectiveness of the inactivated vaccine (254). An SWCNTs-DNA vaccine encoding matrix protein provides a good protective effect against spring viremia of carp virus by intramuscular vaccination (246). Ramírez-Paredes et al. (2019) (247) developed an autologous whole-cell inactivated vaccine to effectively protect red Nile tilapia from *Francisellosis* by intraperitoneal injection. Xu et al. (2019) (153) developed an effective inactivated vaccine against *Vibrio harzianus* in turbot. They found that the vaccine not only induced humoral immunity but also enhanced the innate immune response to provide long-term effective protection by intraperitoneal injection with different concentrations of the vaccine in turbot. The commercial use of the *Yersinia ruckeri* vaccine without adjuvant is possible, but efficiency is insufficient, wherein the use of aluminium hydroxide is harmless and does not cause complications (255).

### 4.2 Immersion immunization

Immersion immunization is a unique way of vaccination for aquatic vaccines. Antigens can be uptaken by mucosal tissues such as gills, skin, lateral line and gastrointestinal tract, which can quickly and effectively activate the mucosal immune system of fish, and then spread to the peripheral blood, kidney and spleen and other systemic immune tissues through blood circulation to generate a systemic immune response (256). Its advantages are that it causes less mechanical damage and stress stimulation to fish bodies, is easy for herd immunity, has less labour intensity, and less time-consuming immunity. It is suitable for fry and smaller fish. The application of immersion vaccine has been reported in many fish, such as *Anguilla japonica* (257), Atlantic cod (258), grouper (259), Crucian carp (260), and rainbow trout (261). Immersion immunity mainly includes Direct immersion (DI), Hypertonic immersion (HI), Flush and Spray, etc (262). DI involves transferring fish to water containing the vaccine for some time and then returning them to the breeding pond. This method is simple and suitable for sterilization vaccines and attenuation vaccines and causes less stress to fish bodies. Therefore, it can improve the uptake of antigens by prolonging the immersion time, thus improving the effect of immersion immunity and reducing the cost of vaccines (263, 264). Many studies show that the immune protection of direct immersion immunization is low, and needs to be strengthened to achieve the ideal immune effect (265). The hyperosmotic method is to soak the fish in 3% ~ 5% salt solution for 5 minutes, then move the fish herd to the vaccine solution for immersing, to force the antigen to infiltrate into the fish by the change of osmotic pressure (248, 266). Ultrasonic treatment can increase the permeability of biological tissues such as skin and muscles, and the number of antigens entering a fish’s body can be significantly increased by ultrasound in spray (267, 268). Although hypertonic and spray methods can improve the efficacy of immersion vaccines, their application is limited by stress on fish bodies. The uptake of antigen by immersion is limited, so the immune effect of immersion on fish is lower than that of injection immunization. Moreover, the immersion environment has a great influence on the vaccine effect, and it is difficult to control the amount and time of immersion. Immersion usually requires a large amount of vaccine, and in practice, vaccine cost must be a primary consideration. In recent years, many studies have focused on how to improve the effect of immersion immunity using different methods. Examples are booster vaccination, administration of immunostimulants/adjuvants, use of delivery vehicles, preconditioning by physical methods, use of novel live attenuated vaccines and DNA vaccines, etc (269–271). Mucoadhesive polymers are widely used in drug delivery and can also be developed and utilized as vaccine carriers in immersion immunization (272–274).

### 4.3 Oral immunization

Oral administration is considered to be the most practicable form of immunisation for aquatic vaccines and is suitable for a wide range of vaccine types including inactivated, live attenuated and nucleic acid vaccines. Compared to injectable vaccination, oral administration is not restricted by the size and age of the animal, can reduce stress and mechanical damage to the animal, can provide some savings in labour costs and meets animal welfare requirements (275). At present, aquaculture is most intensive and factory-based, and oral immunization is more suitable for large-scale farm animals than other immunization methods. However, the development and application of oral vaccines for fish are not yet widespread. The main constraint is that the vaccine is susceptible to destruction by digestive tract enzymes, cannot induce intestinal mucosal immunity and systemic immune responses, and may induce immune tolerance. In addition, the number of orally immunised fish and the dose of vaccine cannot be effectively guaranteed, resulting in a lower immune protection effect than that of injection immunization at present. To improve the immune effect of oral vaccines, it is necessary to avoid naked antigens as much as possible. At present, the common materials used to encapsulate vaccines include alginate, chitosan, liposomes and other polymeric microspheres and biofilms (275–278). It has been shown that DNA vaccine can improve fish defence against disease, but the immune effect of orally administered naked DNA vaccines is not ideal, instead, the immunological effect of the vaccine can be significantly improved after encapsulating with sodium alginate (279–281). In addition, the recombinant subunit vaccine can also be administered orally after being encapsulated. Caruffo et al. (2016) (282) used brewer’s yeast to simultaneously express the surface protein of the Hemagglutinin-esterase virus and the F protein of the salmon anaemia virus (ISAV) and encapsulated it in a cationic polysaccharide matrix capsule. The salmon obtained a survival rate of 66.7% after feeding. Biofilm (BF) are Extracellular polymeric substances (EPSs) attached to the surface of microorganisms and serve as a protective barrier for microorganisms to enhance their tolerance to environmental stresses, such as desiccation, osmotic pressure, UV radiation, disinfection and antibiotic therapy (283). The biofilm can protect the antigen components of the vaccine from the destruction of the digestive fluid as it reaches the gut lymphoid tissue, allowing the oral vaccine to perform its proper immune function (284, 285). Due to the high cost of the encapsulated material and its large amount of use, it is unrealistic in practical application. And the mechanism of immune activation in the animal intestinal mucosa is not clear, some materials may cause non-specific reactions and reduce vaccine effectiveness (286, 287). Therefore, in recent years, the research of another biological carrier vaccine has become the focus of oral vaccine research. Genetic engineering allows the expression of exogenous antigen components through non-pathogenic microorganisms. Reported biological carriers include insects (halogen larvae, beet noctule, silkworm pupae, etc.), probiotics (lactobacillus, bacillus subtilis, yeast, etc.), plants (Chlamydomonas, fishy algae and potatoes, etc.) and viruses (288). Compared to traditional vaccines, biocarrier vaccines are more effective at presenting antigens and antigen selectivity (205, 289). Different vector vaccines can be prepared according to different antigen properties, or insert multiple exogenous antigens into the same vector vaccine to achieve the effect of one vaccine against several diseases. It is noteworthy, however, that biological vector vaccines often receive interference from vector-derived antibodies during booster immunization, and that the components of vectors such as viruses can also pose a potential threat to the host. Therefore, the selection of a suitable biocarrier for the antigen and the improvement of the safety and applicability of biocarrier vaccines have become prerequisites for the mass production of biocarrier vaccines.

Currently, the immune response to oral vaccines is still different from that of vaccines administered *via* injection, and in some cases, it is just half as strong. Further in-depth research is required on several topics, including the immune mechanism of oral vaccines, in particular the immune effect on the intestinal mucosa, the handling of antigens during vaccine production, the effective dose, the type of vaccine coating material, carrier, and adjuvant, the form of the vaccine, as well as the duration of immunization, the frequency of immunization, and the physiological developmental stage of the experimental fish.

## 5 Mechanism of aquatic vaccines

Teleost fish possess both primary and secondary lymphoid tissues. Primary lymphoid tissues include the thymus, where T-cell development occurs, and the head kidney, which performs hematopoietic functions similar to mammalian bone marrow. Secondary lymphoid tissues include the spleen and mucosa-associated lymphoid tissue (MALT). There are four kinds of MALTs, nasopharynx-associated lymphoid tissue (NALT), gill-associated lymphoid tissue (GIALT), skin-associated lymphoid tissue (SALT), and gut-associated lymphoid tissue (GALT), which contain specific adaptive immune systems (290, 291). In general, teleost MALT does not have organogenic lymph nodes or lymphatic aggregates but consists of a diffuse network of myeloid and lymphoid cells (292). However, there are some T lymphocyte aggregates within the GALT, called interbranchial lymphoid tissue (ILT), despite the lack of a complete regional growth centre for B and T cells (293). At present, the mechanisms of antigen uptake and antigen presentation in the teleost MALT are unclear. In all organisms, the mucosal surface is the main site of entry for pathogens, while the mucosal surface is covered with an immune-enhanced mucus protective layer, which acts as the first line of defence against pathogens. Fish mucus contains a variety of immune-related factors such as lysozyme, defensins, immunoglobulins, etc (294). Teleost fish also possess an adaptive immune system that relies on somatic recombination of germline-encoded VDJ fragments to produce a large number of antigen receptors expressed on T and B lymphocyte membranes (295). Studies have shown that teleost MALT has a large number of antigen-presenting cells in mucosal sites, including dendritic cells (DCs), macrophages, IgT/Z^+^ B cells and granulocytes, all of which have been shown to have antigen uptake functions (296–298).

### 5.1 Mechanism of injection immunization

The immune organs of fish are simpler, mainly including the thymus and spleen, as well as the anterior kidney, which functions like bone marrow and lymph nodes, and the scattered lymph-like tissues containing lymphocytes, monocytes, macrophages, granulocytes, platelets, mast cells, and non-specific cytotoxic cells (299). Fish have mammalian-like T and B lymphocytes. Different subpopulations of fish T cells have not been well characterized, but homologs of CD8 and CD4 have been reported, while B lymphocytes were also found to have phagocytic activity (300, 301). Fish MHC I, TCR and TCR co-receptor CD8 are homologous to mammals, suggesting similarities in the antigen presentation process, i.e., antigens presented by MHC are recognized by TCR and activate immune responses. In addition to TCR-specific recognition of MHC I/II, natural antigens are also recognized directly by B lymphocyte receptors. Currently, it is generally accepted that the specific immune response in fish is the basis of Injectable immunization. When the antigen invades the fish, it is presented by antigen-presenting cells (APCs) to T cells, and then presented to B cells by T cells, leading to B cell activation, proliferation, and differentiation into plasma cells and memory cells, which eventually produce a large number of antibodies, resulting in a specific immune response. Antibodies are the main functional factors of the fish immune system, and in recent years four classes of Ig have been identified in fish, namely IgM (302), IgD (303), IgZ/T (304, 305) and IgM-IgZ (306). Injection immunization mainly causes the immune system of fish to produce IgM. After intraperitoneal immunization, mucosal IgM antibodies and pIgR responses are triggered, and the pIgR produced by local plasma cells in the lamina propria (LP) mediate the IgM-antigen complex through the intestinal epithelium into the intestinal mucus (307). Antigens being injected intramuscularly can evoke a local adaptive immune response, however, the mechanisms regarding the dynamics and transport of antigens after injection have not been elucidated. There are differences in the immune response of various organs caused by injecting immunity, mainly inducing a response in the systemic immune organs such as the spleen and kidney, and a weaker immune response in the mucosa tissues such as gill, skin and intestinal (308). Interestingly, we found that the difference in antigen uptake after intraperitoneal and intramuscular injection followed the same pattern: the highest antigen uptake was in the spleen and head kidney, followed by blood, liver, and gills, and the least in the hindgut, muscle, and skin. However, the peak antigen uptake in the spleen, head and kidney, liver, blood, and hindgut was significantly higher in the intraperitoneal injection than in the intramuscular injection. The peak antigen uptake in skin and muscle was significantly higher in the intramuscular injection (309). Moreover, the expression of antigen presentation-related genes (CD4-1, MHC IIα, CD8α, MHC Iα) in the spleen, head kidney, liver and hindgut of flounder, and the proportion of sIgM^+^ cells in the spleen and peripheral blood were higher in the intraperitoneal injection group than in the intramuscular immunization group (310, 311). Both cellular immunity (including cytotoxicity) and humoral immunity are activated by nucleic acid vaccination. Cellular immunity is primarily initiated by antigen-presenting cells (APCs) such as macrophages and dendritic cells (DCs) (312, 313). DNA motifs and mRNA transcripts express immunogenic proteins in fish that simulate infection by intracellular pathogens and allow the presentation of exogenous antigenic peptides on the APC surface *via* major histocompatibility complex (MHC) class I. Subsequently, APC at the site of administration may trigger immune cells (314, 315). Antigen-presenting cells can also absorb soluble antigens released from transfected myoblasts (or other cells) or in the presence of antigenic apoptotic vesicles, delivered to MHC class II molecules on the cell surface. Antigenic peptides in MHC class I and MHC class II are recognized by the T cell receptor (TCR), which activates CD8^+^ T cell (cytotoxic T cells) and CD4^+^ T cell (helper T cells) responses, while the adaptive humoral immune response is manifested by B lymphocyte activation and antibody production. The unmethylated CpG motif is identified by the vertebrate immune system as a “foreign” and “dangerous” signal. For the vectors of nucleic acid vaccine derived from bacterial and viral DNA, which carries unmethylation CpG motif can be identified by the body as a pathogen-related molecular pattern, activating the macrophage, and B cells to induce the humoral immune also can directly activate monocytes, macrophages and dendritic cells to secrete Th1 cytokines to induce a cellular immune response (316, 317). In addition, CpG motifs can be recognized by Toll-like receptor 9 (TLR9) to activate leukocytes by the uptake of DNA into lysosomes containing specific receptors (318, 319). In addition, humoral factors such as complement, cytolysin, interferon, lectin, etc. that mediate fish’s non-specific immunity are important ways for fish bodies to resist the external environment. While macrophages were found to play the phagocytic function of non-specific immunity as well as phagocytosis, processing and presentation of antigens, indicating that specific and non-specific immunity in fish complements each other in the process of injection immunization. Studies have found that phagocytic cells similar to mammalian M cells are distributed in the hindgut of fish. After intraperitoneal injection, antigens are recognized, processed and presented by these cells, thus causing immune responses in fish.

### 5.2 Mechanism of immersion immunization

The gills and skin mucosa not only have non-specific immune functions but also have specific immune response functions (187, 320). The mucosal tissues and their secreted mucus contain abundant non-specific immune factors such as hydrolase, enzyme/transfer factor, chitin, C-reactive protein, lectin and specific antibody proteins, which together constitute the first line of defence against the invasion of pathogenic microorganisms in fish (321, 322). It was found that there were a certain number of T and B lymphocytes, mucous cells, cystic cells and antibody-secreting cells in the skin of fish. Similarly, various immune cells such as lymphocytes, macrophages, goblet cells and neutrophils were found in gill tissues. The level of antibodies and the number of antibody-secreting cells on the surface of gills, skin and intestinal mucosal tissues were significantly increased after vaccine immersion, which indicates immersion immunization can cause an obvious immune response (323, 324). Numerous studies have now shown that mucosal immunity can produce a local immune response independent of the systemic immune response. Immersion immunity mainly induces a mucosal immune response in fish, while systemic immune response is weaker and later than mucosal immunity. During immersion immunization, antigens are first taken up by mucosal tissues such as gills, skin and intestine, which activate lymphocytes in mucosal tissues to produce antibodies. Through blood circulation, antigens are transported to tissues such as the spleen and kidney and activate systemic immune responses (256). Antigen uptake was positively correlated with immersion concentration in a certain range, and antigen particles on the gills were mainly taken up by phagocytes, while antigen recognition and presentation in the skin were presumably done by macrophages (325). Three immunoglobulin isotypes (IgM, IgT and IgD) were detected in the gills, skin and nose of fish, with IgT being the predominant functional Ig isotype in these mucosal tissues and the strongest response of IgT in immersion immunization (326, 327). Some researchers believe that gills have an independent immune response, because MHC II -positive cells were detected in a variety of fish gill tissues, and found that the proportion of ASC on the gills was significantly higher than that in peripheral blood of immersion immunized fish, and the number of antibody-secreting cells at the different time showed a kinetic response pattern (328–330).

### 5.3 Mechanism of oral immunization

Current studies have shown that oral immunization can produce both local mucosal immunity and systemic immune responses, but the intestinal mucosal immune response is predominant (331). The possible mechanism is that the macromolecular antigens were swallowed by the hindgut epithelial cells, and the small molecule soluble antigens were infiltrate the blood through the intestinal mucosal cell gap. While large molecule particle antigens are the first uptake by macrophages into the cells after processing, then transported to the relevant lymphatic tissues or cells, then transported to the circulatory system, and finally transported to the lymph nodes *via* blood or lymphatic fluid to produce the relevant immune response (332). A large number of lymphocytes, mainly including macrophages, granulocytes and plasma cells, are distributed in the entire intestine of fish, especially in the hindgut (333, 334). It is now generally accepted that the hindgut is the main site of antigen uptake and processing in fish and that antigens entering the hindgut are taken up by macrophages to induce an immune response in the fish (334, 335). Companjen et al. (2005) (336) found lymphoid tissue in the gut of *Dicentrarchus labrax*, and the number of lymphocytes showed an obvious numerical gradient from the digestive tract to the anus. This phenomenon suggests that the hindgut produces a stronger immune response than any other part of the intestine, and it is presumed that oral vaccines work mainly in the hindgut. The intestinal mucosa layer of fish can be divided into two layers, the epithelial layer and the lamina propria, in which granulocytes, macrophages and other leukocytes and Ig^+^ cells mainly exist in the lamina propria of the intestinal mucosa layer, while a large number of Ig^-^ cells also exist in the epithelial layer of the mid and hind intestine (337, 338). In addition, T cells are distributed in the epithelial layer of the intestinal mucosa, while B cells are distributed in the lamina propria of the intestinal mucosa to participate in the mucosal immune response. Mucosal immunoglobulins (e.g., IgT or IgZ) are relatively new findings in fish immunology. In rainbow trout intestine, IgT^+^ and IgM^+^ lymphocytes account for approximately 54% and 46% of the total B-cell population, the IgT/IgM ratio is higher in intestinal mucus than in serum, and the percentage of bacteria phagocytosed by IgT (48%) is significantly higher than that of IgM (24%), these studies suggest that IgT is the major immunoglobulin in intestinal mucus (339). In orally vaccinated rainbow trout intestine, IgM^+^ and IgT^+^ cells were found to be distributed throughout the intestine, with IgM^+^ cells located mainly in the lamina propria (LP) and IgT^+^ cells localized mainly as intraepithelial lymphocytes (IEL), and a significant increase in the number of IgM^+^ and IgT^+^ IEL was observed in the pyloric cecum region. Not only that, the authors found that B cells responsive to vaccination were detected in adipose tissue in the digestive tract, suggesting that these cells surrounded by adipocytes also play a role in mucosal defence (340). Oral tolerance is a low response to fed antigens and a result of inhibition of cellular and/or humoral immune responses, a phenomenon also prevalent in fish (341, 342). Many factors, including excessive antigen dose, inhibition of Treg production, repeated administration, low temperature, antigen type, and genetic effects, are prone to induce tolerance in fish (343, 344). Decreased antibody response after repeated exposure to antigens may be associated with the induction of upregulation of FoxP3, TGF-β and IL-10 (345).

## 6 Aquatic vaccines adjuvants

In recent years, immunization control has become an important initiative for aquatic animal disease control, and there has been rapid development of aquatic vaccines and more diversified vaccine types. Traditional inactivated vaccines and emerging recombinant vaccines have problems with high antigen dosage, short duration of immunity, and low immune efficacy, and cannot achieve the desired immunization effect when used alone. An adjuvant is a non-specific immune enhancer that is used before or simultaneously with an antigen to enhance the body’s immune response to the antigen or to alter the type of immune response. The use of adjuvants can reduce the immunization dose, lower the cost of vaccines, reduce vaccine irritation, enhance the body’s immune response to antigens, prolong the protection time, etc. The ideal adjuvant enhances the effectiveness of the vaccine, is free of side effects, and is easily available and universally applied. The development of new aquatic vaccines requires new adjuvants to improve the effectiveness of vaccine use. An appropriate adjuvant enables an effective vaccine to produce efficient immune protection by different vaccination methods, such as the Fno vaccine, which can also improve the protection of Nile tilapia by oral administration with an oral adjuvant (346). New adjuvants should be studied for vaccine type, mode of action and carrier, immune efficiency, the durability of immune effect, and toxicity of the adjuvant itself, to match vaccines better and improve immune protection. Available evidence suggests that adjuvants may generate an immune response in a variety of ways, such as the sustained release of antigen at the injection site and recruitment of immune-related immune cells, upregulation of cytokines and chemokines, promotion of antigen uptake and proliferation of antigen-presenting cells (APCs), and activation of inflammatory responses (347). Moreover, the majority of adjuvants are currently on the market for injectable vaccines, while immersion and oral vaccines are less available, which limits the development of immersion and oral vaccines to a certain extent. Compared with injectable vaccines, immersion and oral vaccines have the advantages of convenience, low stress and low cost, so the application prospect of immersion and oral vaccine adjuvants is promising. Based on their mode of action, adjuvants can be categorized broadly into antigen-presenting and immune-enhancing types.

### 6.1 Antigen-presenting adjuvants

Antigen-recurrent adjuvants include aluminium salts, oil emulsions and granules, which are usually presented to the immune system in the form of endocytosis, pinocytosis, and membrane fusion to promote an immune response to antigens. Among them, aluminium adjuvant has a powerful delivery effect and is widely used in many types of vaccines. Aluminium adjuvants can enhance the expression and duration of major histocompatibility complexes (MHCs) on the surface of dendritic cells, promote antigen delivery to intracellular antigens, and thus produce adaptive immune responses (348, 349). Aluminium adjuvants cause less tissue damage and few adverse immune reactions and hypersensitivity reactions and are considered safe adjuvants. Currently, most of the vaccines used in production are aluminium-adsorbed vaccines (350).

Oil adjuvants are the most commonly used adjuvants in aquatic vaccines, and almost all salmon are vaccinated against oil adjuvants. Of these, the complete Freund’s adjuvant (CFA) is composed of heat-inactivated mycobacteria and mineral oils containing surfactants. The adjuvants act as carriers and reservoirs for the presentation of antigens in animals after injection, allowing slow release of antigens and continuous stimulation of strong Th1 and Th17 reactions in animals through MyD88 pathways to produce higher immunity (351). CFA is the most widely used adjuvant (352). CFA causes fish in long-term antigen environmental stimulation, although it improves antibody levels, it also has side effects associated with inflammatory response, and usually causes damage in injection sites such as the pyloric blind sac, spleen, muscles, oesophagus, etc., affecting the growth of fish (353). Incomplete Freund’s adjuvant (IFA) does not contain mycobacteria, can improve cell phagocytosis, promote leukocyte infiltration and cytokine production, and produce long-lasting immune protection, IFA also has serious toxicity (354). The MONATINE series of mineral oil-based adjuvants developed by SEPPIC is easy to emulsify, low viscosity, stable and safe to absorb, and widely used in fisheries vaccines (355).

Microparticle is a new type of delivery vector widely used in vaccine adjuvants, including virions, nanoparticles, polymers, etc. Antigens such as polypeptides, proteins and DNA are transported inside particles to lymphatic organs through covalent binding or physical burial and are released continuously through void diffusion and particle degradation. At present, covalent coupling of antigen and micro grain has become a new technique, which can reduce antigen dose, protect antigen integrity, and improve antigen stability and immune efficiency (356–358). Studies have shown that the micro granule adjuvant effect is closely related to micro granule size, and the smaller the particle, the higher the immune efficiency. For example, microparticles often induce humoral immunity and nanoparticles mainly produce cellular immunity (359–364).

Polylacticacid (PLA) is a biodegradable histocompatibility polymer. PLAs can quickly reach the cytoplasm by phagocytic action or clathrin-mediated endocytosis causing an early immune response (365). As an adjuvant, polylactic-co-glycolicacid (PLGA) can cause cellular and humoral immunity and improve antigen delivery. It also has the advantages of good histocompatibility, biodegradability and non-toxicity. In an application of an outer membrane protein (OMP) based vaccine against *A. hydrophila*, the PLGA adjuvant showed a better vaccine immune effect compared with the IFA adjuvant (366).

### 6.2 Immune-enhancing adjuvants

Immuno-enhanced adjuvants indirectly cause a congenital immune response, or directly activate a congenital immune response, mainly by activating the pathogen recognition receptor (PRRS) (367). PRRS is mainly divided into membrane receptors (including Toll-like receptor, TLR, C-type lectin, etc.), intracellular receptors (including NOD-like receptor, RIG-I-like receptor) and secretory receptors, which are expressed in a variety of immune cells, to activate congenital immune responses by identifying PAMPs, or by stimulating the secretion of inflammatory cytokines and activating antigen-transitive cells, thus establishing an adaptive immune response. A total of 17 different types of TLR genes are found in fish, far more than in mammals, and immune enhancers are expected to play an important role in aquatic vaccines.

Saponins are natural steroids or terpenoids, widespread in animals and plants, low-grade marine animals and bacteria. Saponins can stimulate the secretion of specific antibodies, enhance the phagocytosis of macrophages and respond to exogenous antigens by cytotoxic t-lymphocytes (CTL). QS-21 is a saponin extracted from a soap tree that enhances the secretion of Th1 cytokines (IL-2 and IFNγ) and IgG2-a. In aquatic, saponins can enhance the non-specific immunity of shrimp and stimulate fish growth, but high-dose saponins can easily cause severe tissue damage in fish when using Intraperitoneal injection (368, 369). In the study of immersion immunity, it is shown that saponins can significantly improve the delivery of vaccine antigens in the skin and other tissues, trigger the inflammatory response, and improve the effect of non-specific immune levels in fish (370). Usually, the longer the immersion immunization time, the better the immunization effect and the adjuvant can accelerate the absorption of antigens, shorten the immunization time and reduce the stress on the fish body (371). Therefore, the application of saponins as an immersion vaccine adjuvant is of great value, but it is necessary to solve its instability in the water phase.

Cytokines are a class of small molecular proteins secreted by immune cells and non-specific immune cells, which play an important role in pro-inflammatory response and immune system regulation. The structural function of fish cytokines is similar to that of mammals, including IL, IFN, tumour necrosis factor and chemokine, etc., with good adjuvant effects. Studies have shown that a large number of B cells and cytotoxic T cells accumulate at the muscle site where IFN is injected. Different IFNs have different biological activities, while IFN, as an autoantigen, can maintain long-term adjuvant activity without attack by the immune system (372, 373). Cytokine IL (e.g., IL-8, IL-6) has been shown to upregulate genes associated with inflammatory response, humoral immunity and cellular immunity, improve immunoprotection and maintain long-term vaccine protection (370).

Others such as β-glucan, mannose (374, 375), chitosan, (376, 377)monophosphoryl lipid A (MPLA), flagelloprotein, CpG ODNs (CpGoligodeoxy nucleotides), poly-nosinicopolycytidylicacid, Poly (I-C) (68), insect hemocyanin (378), propolis (379), complement, etc. can also be used as adjuvants. Among them, β-glucan is the best adjuvant currently used in fish oral vaccines (380, 381). *β*-glucan or anisodamine as adjuvant *β*-glucan can enhance the immersion immune efficacy of inactivated CyHV-2 vaccine in gibel carp (382). More work is needed to present propolis as a proper candidate for the development of a natural adjuvant in aquatic vaccines (383). Clinically, MPL has been used in combination with liposomes or emulsions, demonstrating a good adjuvant effect (384). The inactivation vaccine of *E. tarda* in mixed flagelloprotein can significantly improve vaccination protection on flounder (385), which proves that flagelloprotein is an important candidate for the fish vaccine.

## 7 Challenges and prospects for aquatic vaccines

At present, the number and variety of aquatic vaccines with production approvals worldwide are relatively limited, and they cannot fully cover the major diseases of the main breeding species, which is insufficient to support the development of a comprehensive integrated disease immunization and control system. Inadequate supply of efficient vaccine varieties, as well as a lengthy development process, reduce the overall effect of disease immunization and control in the aquaculture production process and reduce farmers’ confidence in the industrial application of vaccines, which harms vaccine promotion and popularization, as well as investment in the fish vaccine industry. Given the current state of fish vaccine development, more research in the field of fish vaccine engineering is required, primarily to address the following technical challenges and accelerate the development of high-efficiency, multiplexed aquatic vaccines.

### 7.1 Research on types of fish vaccine and vaccination routes

Most vaccines are currently administered *via* injection, which is undeniably a very effective way to vaccinate expensive nucleic acid vaccines and subunit vaccines and is practical for the majority of vaccine types. However, given the small size of aquatic animals and the large number of vaccinations, oral and immersion vaccines appear to have more potential. While immersion vaccination necessitates vaccines that are inexpensive and simple to prepare in large quantities, such as whole bacteria-inactivated vaccines, attenuated vaccines, and so on. It is especially suitable for mass immunization of juvenile fish, which have tender skin and easily absorb antigens, whereas slightly larger fish must be vaccinated with the help of adjuvants, penetrants, carriers, and so on to achieve the desired effect, and research in this area is still limited. Oral immunization is the simplest and quickest method of immunization, with a low workload and little stress on the fish, and it is not limited by fish size, species, location, or time, making it a more ideal method of vaccine administration. However, current research indicates that an oral vaccine is less effective than injectable immunization because the integrity of the antigen is destroyed in the digestive tract, resulting in a weaker response from the intestinal mucosal system; second, the mucosal immune system of aquatic animals is not perfect. Nonetheless, antigens absorbed by mucosal tissues are transmitted to systemic immune tissues, resulting in a systemic immune response. As a result, future research on the mechanism of mucosal immunity and the relationship between mucosal and systemic immunity will be required. Furthermore, more detailed research is required on antigen treatment in vaccine production, effective doses, vaccine coating materials, carriers and adjuvants, vaccine forms, as well as the duration of immunization, the number of immunizations, and the physiological developmental stages of experimental fish.

### 7.2 Improve the evaluation system of fish vaccine

Survival of animals after the attack was the most primitive method for evaluating the effectiveness of vaccines. With the advancement of flow cytometry, monoclonal antibody preparation technology, and multi-omics, vaccine efficacy can now be evaluated comprehensively by detecting changes in gene and protein levels, as well as antibody and cellular responses. In general, vaccine evaluation systems have been rapidly developed, but more in-depth studies, particularly at the cellular response level, are required. The investigation of vaccine response at different levels in the animal organism not only evaluates vaccine effectiveness but also promotes in-depth research on immune mechanisms in aquatic animals. More and more immune cell types are being discovered and the role these cells play in the vaccine immunization process should be appreciated. The lack of antibody probes for fish cells has resulted in many more cells being limited in their detection during vaccine immunization. Despite the current high cost of single-cell sequencing, it is undeniably important to assess vaccine efficacy at the single-cell level, as this will aid in the development of efficient vaccines.

### 7. 3 Strengthen basic research related to fish pathogens and diseases

With a global shortage of fishery resources, an increasing number of aquatic animals are shifting from wild to factory farming, resulting in an increase in diseases that is becoming increasingly complex. The complex farming environment has resulted in disease outbreaks, with the majority of infections being conditional pathogenic and multi-pathogenic. Serotyping of fish epidemic pathogens is the foundation for effective vaccine research, and the lack of efficacy of many currently promoted vaccines is directly related to significant mutation of the relevant epidemic pathogens. However, the rapid development of genomic sequencing technology in recent years has made molecular typing and systematic identification of pathogens much easier, which is very helpful for ongoing research on pathogen serology and epidemiological patterns. Furthermore, there is a need to expand research into the mechanisms of pathogenic conditions that cause disease and disease outbreaks. Environmental factors such as temperature, dissolved oxygen, salinity, and nutrient salts may be causative factors of fish disease outbreaks in the “triad” system of pathogen, environment, and host, and an in-depth understanding of the signal transduction mechanism of pathogenic bacteria to related factors can lay a solid theoretical foundation for vaccine development and integrated immune control of diseases.

### 7. 4 Promoting the molecular design of efficient aquatic vaccines using multi-omics technologies

The screening of critical antigens is required for the efficient design of novel live attenuated, nucleic acid, and genetically engineered vaccines. Furthermore, with the rapid development of various omics technologies (genome, functional genome, proteome, and metabolome) and genome editing technologies, the genetic background and genomic information of various pathogens, as well as the dynamic process of pathogen infection, are better defined, allowing for the systematic screening of genetic targets for highly effective vaccines, particularly attenuated targets and safety targets of live attenuated vaccines. Furthermore, in recent years, structural biology has resolved an increasing number of critical protein structures of pathogenic bacteria, allowing for more precise screening and design of antigenic targets, which will greatly facilitate the molecular design of efficient vaccines, the discovery of antigenic epitopes, and the development of subunit vaccines, multiplex vaccines, and so on. There is no doubt that reverse genetics and reverse vaccinology are important directions for future development, and nucleic acid vaccines such as DNA vaccines, live vector vaccines, and mRNA vaccines have emerged as hot spots for viral infectious disease prevention. Synthetic biology should be used in the future to overcome key technical bottlenecks in the design and delivery of nucleic acid vaccines. Simultaneously, the mechanism of action and application potential of nucleic acid vaccines in the immune protection of fish in theropods should be investigated, as should the synergistic effects of different vaccination/delivery routes on systemic and local mucosal immunity.

### 7. 5 Construction of a technological platform for fish vaccine engineering research

Fish vaccine development is a complex systemic project that necessitates additional in-depth research on the immune system of fish, particularly on immune response generation, immune memory, immune tolerance after fish vaccination, and the homeostatic balance and conversion mechanisms of natural, adaptive, and trained immunity. Meanwhile, systematic research on vaccination protocols, disease early warning, animal models, vaccine adjuvants, immune boosters/modulators, therapeutic vaccines, manufacturing, and registration protocols should be strengthened. To promote the efficient development and application of fish multiplex vaccines, a complete and systematic engineering technology platform for aquatic vaccines can be formed. Vaccination can provide complete disease prevention and control coverage, particularly for key fish culture species and major diseases.

In general, there is a lack of a large amount of experimental data and information to support the immune mechanism of vaccine timing, immunization mode, number of immunizations, and time of booster immunization due to imperfect research on the basic immunology of aquatic animals, combined with the large range and variety of species spanned by aquatic animals. The aforementioned factors have severely hampered the development and application of aquatic vaccines. Therefore, it is critical to strengthen basic theoretical research on aquatic vaccines. The structure, function, and biological properties of pathogens, the technical basis and methodology of fish vaccine design and production, and the relationship between the immune effect of fish vaccine and the environment and organism will all be important foundations for fish vaccine development.

## 8 Conclusion

In recent decades, aquatic vaccines have been developed with some success and their application in aquaculture production has become increasingly popular. The promotion of aquatic vaccines can reduce the use of drugs, reduce diseases, protect the environment and improve the quality and safety of aquatic products. Although some aquatic vaccines against bacterial and viral diseases are currently available on the market, due to the numerous serotypes and easy variation of most pathogens, the strains isolated from different regions and fish species vary significantly, resulting in uneven vaccine effects. Parasitic and fungal diseases are causing increasing damage to aquaculture, but there is a serious lack of commercial vaccine. Genetic engineering vaccines consisting of protective antigen genes and a newer generation of nucleic acid vaccines have shown better immunity, but many studies are still in the experimental stage and fewer vaccines are commercially available. Combining vaccines with immunostimulants such as probiotics and herbal additives to improve the animal’s immune effect is an important measure. In addition, important progress has been made in recent years to address disease problems by breeding resistant strains of aquatic animals. On this basis, exploring the functional genes and proteins of resistant strains through gene and proteomics, resolving the differences in animal immune systems, and revealing the mechanisms of pathogenic infestation and pathogen-cell interaction will provide important theoretical guidance for vaccine design. Overall, it is a long process for the aquatic vaccine from research to commercialization, in which the lack of technical content, cumbersome approval procedures, and insufficient promotion efforts are seriously hindering the development of the aquatic vaccine industry. At present, many aquatic vaccine developments are in the clinical trial stage, and it is believed that there will be many commercial vaccines available in the next few years, which will greatly promote the development of the aquaculture industry.

## Author contributions

YD, LM and JC designed the article framework and content. YD and XMH wrote the review content. All authors contributed to the article and approved the submitted version.

## Funding

This work was supported by the National Natural Science Foundation of China (41906107), the Key Laboratory of Marine Biotechnology of Fujian Province (2021MB04), and the Key Scientific and Technological Grant of Zhejiang for Breeding New Agricultural Varieties (2021C02069-1).

## Conflict of interest

The authors declare that the research was conducted in the absence of any commercial or financial relationships that could be construed as a potential conflict of interest.

## Publisher’s note

All claims expressed in this article are solely those of the authors and do not necessarily represent those of their affiliated organizations, or those of the publisher, the editors and the reviewers. Any product that may be evaluated in this article, or claim that may be made by its manufacturer, is not guaranteed or endorsed by the publisher.
